# Combining magnetic resonance imaging with readout and/or perturbation of neural activity in animal models: Advantages and pitfalls

**DOI:** 10.3389/fnins.2022.938665

**Published:** 2022-07-15

**Authors:** Lauren Kosten, Serena Alexa Emmi, Stephan Missault, Georgios A. Keliris

**Affiliations:** ^1^Bio-Imaging Lab, Department of Biomedical Sciences, University of Antwerp, Antwerp, Belgium; ^2^Foundation for Research & Technology – Hellas, Heraklion, Greece

**Keywords:** multimodal imaging, fMRI, neural activity, hemodynamic response, optical imaging

## Abstract

One of the main challenges in brain research is to link all aspects of brain function: on a cellular, systemic, and functional level. Multimodal neuroimaging methodology provides a continuously evolving platform. Being able to combine calcium imaging, optogenetics, electrophysiology, chemogenetics, and functional magnetic resonance imaging (fMRI) as part of the numerous efforts on brain functional mapping, we have a unique opportunity to better understand brain function. This review will focus on the developments in application of these tools within fMRI studies and highlight the challenges and choices neurosciences face when designing multimodal experiments.

## Introduction and state-of-the-art

Functional magnetic resonance imaging (fMRI) is a powerful neuroimaging tool that since its inception in 1990 (Ogawa et al., 1990) has revolutionized human as well as non-human neuroscience and biomedical research. FMRI’s global brain coverage and relatively good spatial and temporal resolution can provide invaluable insights into whole-brain activity and network organization. Moreover, its non-invasive nature without ionizing radiation allow repeated and longitudinal *in vivo* acquisitions in the same subjects and thus evaluation and follow-up of disease progression and/or therapeutic outcome.

Functional magnetic resonance imaging relies on the coupling between neural activity and hemodynamic responses, known as neurovascular coupling (NVC) (Ingvar and Lassen, 1962). In its most common application, fMRI detects the blood-oxygen-level-dependent (BOLD) signal, which reflects changes in the relative levels of oxygenated and deoxygenated hemoglobin driven by changes in cerebral blood flow, blood volume, and blood oxygenation, which are coupled to neural activity by NVC (Buxton and Frank, 1997; Logothetis and Wandell, 2004). Hence, fMRI provides an indirect measure of neural activity. More specifically, neural activity has higher energy demands met by oxidative metabolism and that requires increases in oxygen consumption. The brain’s vascular system responds to this demand by increases in cerebral blood flow (CBF), termed as “functional hyperemia,” and cerebral blood volume (CBV) resulting in a local markedly increased ratio of oxygenated hemoglobin (HbO) over deoxygenated hemoglobin (HbR) (Attwell and Iadecola, 2002). The differences in magnetic properties of HbO (weakly diamagnetic) and HbR (strongly paramagnetic) result in measurable changes in MRI BOLD signal when their ratio is altered allowing the detection of activated brain areas. The capability of neuronal activity to trigger metabolic responses *via* the NVC became clear when autoradiography studies using the 2-[^14^C]-deoxyglucose (2DG) approach had been conducted (Sokoloff et al., 1977). Since then, the NVC has received much attention in the literature and the acquired knowledge has underlined its high level of complexity. Further, thanks to the research conducted by Chance et al. (1962) and Jöbsis (1977), it was shown that neuronal activity is not only able to trigger changes in CBF and CBV but also in oximetric signals detectable *via* optical imaging. The NVC is governed by several mechanisms and signaling pathways that are still far from being understood (Lok et al., 2007). Thus, although a link between neural activity and hemodynamic responses is established, their exact relationship is still an open research query. It is of importance to shed light on these phenomena not only to correctly interpret fMRI data but also to provide a better understanding of pathological conditions such as stroke or neurodegenerative diseases that are characterized, among other features, by the disruption of the NVC (Girouard and Iadecola, 1985; Jonckers et al., 2015). Currently, different fMRI methods are in use, including resting state [rsfMRI, Jonckers et al. (2015)], stimulus-evoked [st-fMRI, Barry et al. (2021)], and pharmacological fMRI [phMRI, Zhao et al. (2021)]. Respectively, these techniques permit the assessment of functional connectivity between brain regions during rest (measured by the correlation between low frequency fluctuations in the BOLD signal) as well as brain activation triggered by sensory stimulation or a pharmacological challenge. Each of these methods pinpoints different aspects of neuronal activity, making it a technique suitable to investigate different aspects of brain function.

**FIGURE 1:**
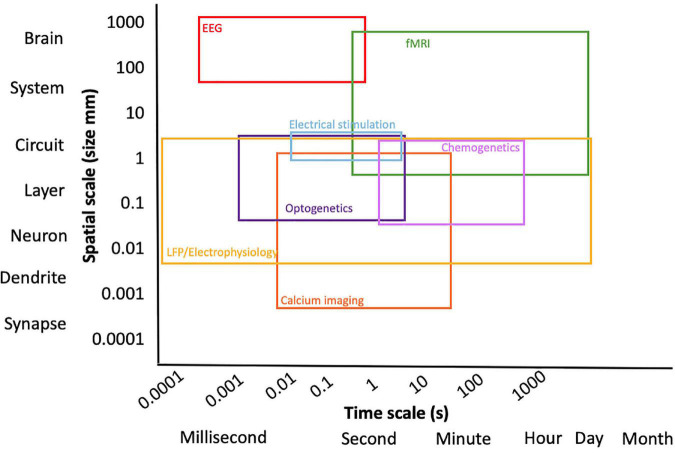
Overview of multimodal techniques expanding the spatial and temporal resolution of the experiment.

In order to obtain full advantage from the application of fMRI, its strengths and weaknesses should be taken into consideration, together with the potential of other neural activity readout and neuromodulation approaches (Figure 1). It is essential to recognize what fMRI can and cannot do (Logothetis, 2008). On the one hand, fMRI is characterized by relatively high three-dimensional spatial resolution of the whole brain, but on the other hand, it is affected by a relatively low temporal resolution, given that it relies on the NVC that is slower in nature (order of seconds) in comparison to neuronal spiking activity that demonstrates millisecond resolution (Logothetis and Pfeuffer, 2004). As previously mentioned, fMRI is only an indirect measure of neuronal activity. The controversy surrounding the BOLD response and the interpretation of fMRI data is mainly related to it being a “surrogate” signal resulting from hemodynamic changes and therefore not a direct readout of neuronal activity (Logothetis and Pfeuffer, 2004).

Combining fMRI with additional readouts of neural activity provides a powerful tool to gain insights into the NVC and the interpretation of fMRI findings. The most widely used probe for neural activity in experimental neuroscience has been electrophysiological recordings (Hubel, 1957; Wiesel, 1959; Rubaiy, 2017). More recently, the advent of novel technologies such as the development of (genetically encoded) calcium imaging dyes with relatively fast dynamics able to follow neural activity, introduced a stark competitor to electrophysiology. During the last decades, there has been a marked shift from electrophysiological approaches to optical imaging in combination with fMRI in the study of neuronal activity and NVC (Scanziani and Häusser, 2009). Each of these techniques provides a different approach in the study of neuronal activity in combination with fMRI and the use of one rather than another mainly depends on the aim of the study and on the type of neuronal activity investigation that has to be done.

This review compares electrophysiological with optical approaches that can be used in combination with MRI imaging. The focus is to provide an examination of the main features of these methods, including their implementation, costs and MRI compatibility starting from electrophysiology and ending with the most recent state-of-the-art optical techniques. By highlighting the pros and cons of each technique, we aim to guide scientists toward the most suitable technique for their goals.

## Coupling the blood-oxygen-level-dependent signal to the electrical neuronal activity: Simultaneous functional magnetic resonance imaging and electrophysiology

Since its inception, electrophysiology has been considered the “gold standard” to probe electrical neuronal activity (Galvani, 1791; Piccolino, 1997, 1998). Its strength relies on its remarkable sensitivity and temporal resolution in investigating, in the order of milliseconds, the electrical features of neurons by means of electrodes made of different materials (glass, metal, silicon). In particular, it provides direct and precise measurements of electrical currents and voltage changes at different scales, from single ion channels to small neuronal populations, with a high signal-to-noise ratio. However, electrophysiology shows disadvantages as well. While it offers high temporal resolution and precision (single neurons can be measured), it has limited spatial coverage, meaning that recordings of electrical signals are limited to neuronal groups and cannot be extended to the whole neuronal network and thus difficult to do whole-brain recordings. At the macroscale, indirect measurement of large areas of the brain’s activity is achieved *via* electroencephalography (EEG), at the mesoscale the development of microelectrode arrays (MEAs) has increased the number of neurons that can be recorded both *in vivo* and *in vitro*. They are however invasive in an *in vivo* setting and limited to the brain region of implantation and most importantly, they potentially record undefined signals coming from the areas surrounding the array (Obien et al., 2015).

The lack of a one-to-one correspondence between spontaneous electrophysiological and hemodynamic measures suggests that electrophysiology might be able to reveal properties of spatial patterns in spontaneous activity that are undetectable with fMRI. For example, the much better temporal resolution of electrophysiology enables researchers to answer questions about networks such as whether one brain area ‘leads’ while another one in the network “follows.” Measuring correlations between spontaneous signal fluctuations, be it in electrophysiology or in fMRI, has grown to be a much-used way to probe brain functional connectivity during rest. These so-called resting-state networks commonly found in fMRI seem to possess analogous electrophysiological patterns over the cortical surface (Schölvinck et al., 2013). At the same time, the wealth of spectral, spatial, and temporal scales afforded by electrophysiological measurements has begun to put the spotlight on the meaning and value of correlation-based concepts such as functional connectivity and functional networks (Schölvinck et al., 2013). To overcome the disadvantages related to both fMRI and electrophysiology and to achieve their maximum efficiency, they can be advantageously combined to shed light on the neuronal basis of the BOLD signal: while the BOLD signal at the basis of fMRI gives an indirect measure of neuronal activity, electrophysiology provides a direct measure of it (Logothetis and Wandell, 2004), hence becoming the starting technique to elucidate the neural basis of the BOLD signal. To help with interpretation of the neural basis of rsfMRI connectivity, Wang et al. (2019) utilized a multi-modal approach including rsfMRI, optogenetic stimulation (more on this in section Optogenetically modulating neuronal activity using light) and multi-depth cortical electrophysiology recording to examine whether and how the somatosensory thalamus contributes to cortical interhemispheric rsfMRI connectivity. They demonstrated that low frequency activities in the thalamo-cortical network contribute to brain-wide rsfMRI connectivity, highlighting the thalamus as a pivotal region that underlies rsfMRI connectivity. Both techniques are however, susceptible to electromagnetic interference, thus compromising each other in terms of signal and data acquisition when used in simultaneous setups. For example, electrophysiology electrodes can distort the magnetic field and jeopardize the MR image quality. Concurrently, the strong magnetic field can hamper the proper amplification of weak electrophysiological signals recorded inside the magnet. As a consequence, several features of the electrophysiology set-up must be modified to guarantee its functioning in the MRI scanner without compromising the quality of each measurement, therefore demanding a series of technical adaptations such as the use of nonmagnetic electrodes.

**FIGURE 2:**
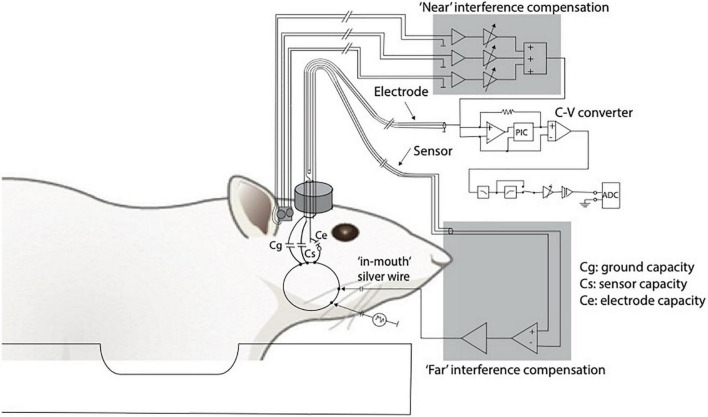
Adapted from Logothetis et al. (2001). Diagram of the recording and compensation circuitry. The animal can be conceived as being capacitively connected to any metal contact, including connections to ground (Cg), sensor (Cs), and electrode (Ce). Because of the finite animal-to-ground capacity, a fraction of the interference currents flow through the electrode. To compensate for such currents, a sensor is built into the electrode holder (current sensor). Interference originating near the electrode tip or within the electrode holder and the cables is compensated for by using three small, orthogonally oriented identical coils positioned near the electrode (magnetic field sensor). C-V, current to voltage converter.

Logothetis and colleagues have played a major role in the development and improvement of these adjustments. One of these is the establishment of an interference compensation system able to compensate the interference to the electrophysiological signals generated by the alternation of the field gradients used for image generation during echo-planar imaging (EPI) (Oeltermann et al., 2007; Figure 2). Their research that sheds light on the neural origin of the BOLD signal *via* simultaneous electrophysiological recordings of local field potentials (LFPs) and spiking activity of neurons of the primary visual cortex and fMRI measurements in anesthetized non-human primates (*Macaca mulatta*) while visually stimulated using contrast gratings is considered fundamental for our understanding of the relationship of BOLD with neural activity (Logothetis et al., 2001). With the obtained results, they provided overwhelming evidence that the BOLD signal strongly correlates with the underlying LFPs, which are more accurately described as a combination of post- and pre-synaptic activity at multiple neurons (“peri”synaptic). This study had a considerable impact because, for the first time, it was demonstrated by means of electrophysiology and fMRI that a spatially localized increase in the BOLD signal directly and monotonically reflects an increase of the neural activity. As such, the BOLD signal is more closely related to LFPs than the spiking activity. The same has been demonstrated when investigating the coupling between the BOLD signal and electrophysiological recordings in a different brain area of anesthetized macaque monkeys: the motion-sensitive area MT in the visual cortex (Lippert Michael et al., 2010). Later, consistent with these results, other studies confirmed that the BOLD signal is better predicted by LFPs, even in case of pharmacological dissociation of spiking activity and LFPs (Rauch et al., 2008), as well as in alert and behaving experimental conditions (Goense and Logothetis, 2008). In fact, it was necessary to investigate if the same results could be reported in alert, awake animals because of the well-known effect of anesthesia on both neuronal activity and vasculature, consequently affecting the NVC (Williams et al., 2010; Masamoto and Kanno, 2012; Jonckers et al., 2015).

Pan et al. (2010) approached the issue of electrodes distorting the MRI images and the MRI acquisition inducing noise in the electrical recordings through the use of glass microelectrodes rather than metal and implementation of a noise removal algorithm for the electrophysiology data in a rat model. Their modified method of electrode implantation minimizes influence on the image quality, and successfully combined the strengths of functional brain imaging with electrophysiology. Jaime et al. stated that their use of intracortical implantation of a multichannel microelectrode array resulted in minimal distortion and signal loss in fMRI images inside a 9.4T MRI scanner. Due to nanomanufacturing advances permitting the production of MRI-compatible microelectrode arrays with 16 or more channels, multimodal research is widely extended. The methodology even permits longitudinal data collection of LFP and rsMRI (Jaime et al., 2018; Zaldivar et al., 2018). Other successful attempts at MRI compatible recordings include the use of carbon fiber electrodes placed on the skull or surface of the brain (Opdam et al., 2002; Austin et al., 2003; Nersesyan et al., 2004; Mirsattari et al., 2005; David et al., 2008) calomel electrodes anchored to the skull (Brinker et al., 1999), platinum wire electrodes covering the scalp (Sumiyoshi et al., 2011), saline-filled (Canals et al., 2009; Moreno et al., 2016) or carbon fiber-threaded (Shyu et al., 2004) glass micropipettes inserted into the brain. Duffy et al. took it a step further by developing carbon fiber optrodes (optical fiber and electrode hybrid devices), which can be utilized in chronic longitudinal studies (Duffy et al., 2015). It is evident that electrodes often cause susceptibility artifacts and could compromise fMRI outcomes. Some studies often show considerable artifacts nearby the electrodes (Walton et al., 2021). The use of tungsten microwires was an initial improvement (Chao et al., 2014; Lai et al., 2015; van den Berge et al., 2017), and further advanced with for example graphene fiber electrodes (Zhao et al., 2020) and advanced imaging sequences to obtain susceptibility artifact free fMRI data such as the 3D radial MRI pulse sequence “Multi-Band SWeep Imaging with Fourier Transformation (MB-SWIFT)” (Lehto et al., 2017).

Taken together, these findings and methodological adaptations allow to compare results coming from human fMRI studies with plenty of electrophysiological data obtained not only from anesthetized animals but also from animals in alert and behaving conditions (Keliris et al., 2007, 2019).

While adaptations for MRI compatibility are not widespread, there are excellent commercial *in vivo* recording systems available such as Smart Ephys to record, amplify, and analyze signals from freely moving or anesthetized animals (Multi Channel Systems MCS GmbH, 2020). Similarly, commercially available probes such as NeuroNexus or ATLAS Neuroengineering are widely used in literature, however, these systems still need adaptations for multimodal use. When it comes to commercially available MRI compatible systems, Blackrock has a unit named MagRes, which is an MRI compatible headstage that will allow small animal researchers to directly correlate neural activity with hemodynamic changes detected on MRI for awake and responsive subjects (Blackrock Neurotech, 2021).

In terms of translation to human studies, EEG and fMRI are two of the fundamental non-invasive methods for identifying brain activity. Multimodal methods have sought to combine the high temporal resolution of EEG with the spatial coverage of fMRI, but the complexity of this approach is still in need of improvement. Nguyen et al. (2018) recently described a method for better EEG and fMRI integration using a spatiotemporal fMRI constrained source analysis method. Similarly, Klein et al. (2015) published a study in 2015 focusing on the influence of the EEG on morphometric measurements of T1-weighted MRI data, recommending against using the structural images obtained during simultaneous EEG-MRI recordings for further anatomical data analysis, despite the benefit of time and as long as no final solution is found for correcting the artifacts caused by the EEG setup.

## From electrons to photons: Magnetic resonance imaging combined with optical imaging as a readout

The scientific community is experiencing a replacement of electrophysiological techniques by optical techniques, mainly because optical imaging offers greater advantages in probing neural activity in terms of both spatial resolution and target specificity by means of genetic approaches (Luo et al., 2008). While electrophysiology is spatially limited (only able to record electrical signals from neurons within a circumscribed brain region surrounding the recording electrodes), optical imaging allows simultaneous recordings from a whole neuronal population of interest at high spatial resolution. Contrary to electrophysiological recordings, optical imaging allows to record neuronal activity without invasive contact with the tissue because light can be transmitted through. Most optical imaging methods make use of (fluorescent) reporters to record neural activity, which, in the last decade, have been subject to continuous improvements. Ultimately, optical approaches have gained use over electrophysiological approaches, revolutionizing research into neuronal activity. Optical measurements of membrane voltage [e.g., *via* voltage-sensitive dyes (VSDs) or *via* genetically encoded voltage sensors (GEVIs)] is however, limited by averaging. Consequently, the detection of single action potentials through optical reporters is still challenging. It is also possible to perform label-free optical imaging of the membrane potential, avoiding the need for exogenous chromophores. However, this technique is still at an early stage, though first demonstrated 70 years ago, and is challenged by the relatively small signals generated by the intrinsic optical properties (Lee et al., 2019). Instead of membrane voltage, intracellular calcium changes can be monitored using fluorescent calcium-sensitive dyes as an indirect measure of neural activity (see Calcium signal as a readout of neuronal activity: Magnetic resonance imaging and fiber-optic calcium recordings). Another complementary technique is intrinsic (signal) optical imaging, which allows scientists to indirectly record cortical neural activity by measuring hemodynamic changes in the brain, and is thus based on the NVC like fMRI (Morone et al., 2017). This method also avoids the need for a fluorescent reporter and there is no need for a cranial window or thinning the skull in small animals. However, these intrinsic signals are much weaker than changes measured through the skull with calcium indicators and have lower spatial resolution. The temporal resolution is limited by the hemodynamic response (Juavinett et al., 2017).

For simultaneous readouts with functional MRI, fiber photometry is ideally suited. An optical fiber can guide the laser beam into the MR bore, while the signal is detected by equipment placed outside of the magnetic field, so that the two methods do not interfere with each other. An array of fibers can be implanted so as to target multiple brain regions of interest (Sych et al., 2019). An important pitfall to be aware of however, is potential heating caused by the laser. This heating can impact the MR signals causing T1 and T2* changes (Christie et al., 2013). Schmid et al. executed control experiments to rule out heat-induces apparent BOLD signal upon stimulation with blue light and propose this may be used as a standard control experiment in opto-fMRI. Occurrence of apparent BOLD is dependent on the energy deposited in the tissue, which is determined by the intensity, duration, and frequency of the stimulation pulses (Schmid et al., 2017). Further things to consider are light propagation in tissue becoming diffusive and therefore losing spatial information (Ioanas et al., 2022b), and correction for absorption of fluorescence light by blood (Zhang et al., 2021).

Opto-fMRI of excitatory vs. inhibitory neurons has also deepened our understanding of BOLD itself. It is assumed that the BOLD fMRI response reflects changes in excitatory neural activity. However, the contribution of inhibitory neurons to BOLD fMRI is unclear. The group of Seong Gi-Kim has published some interesting work in this regard. They selectively modulated inhibitory and excitatory neuronal activity in the somatosensory cortex by 20-s optogenetic stimulation of VGAT-ChR2 and CaMKII-ChR2 mice, respectively, to provide insights into the neurovascular coupling of excitatory and inhibitory neurons and the interpretation of BOLD fMRI signals (Moon et al., 2021). One of their other studies provides useful insights into how perfusion signals of different epileptic states are related in terms of NVC by conducting real-time *in vivo* measurements of cerebral blood flow (CBF), vessel diameter, and excitatory and inhibitory neuronal calcium signals during recurrent focal seizures (Lim et al., 2021).

### Calcium signal as a readout of neuronal activity: Magnetic resonance imaging and fiber-optic calcium recordings

Calcium is a reporter of neuronal activity: changes in intracellular calcium concentration reflect changes in voltage membrane (Grienberger and Konnerth, 2012). Calcium is an important second messenger in eukaryotic cells and it is of central importance to mammalian neurons (Brini et al., 2014). In fact, it plays a crucial role in several neuronal mechanisms at different levels: presynaptically, calcium influx determines the release of neurotransmitter packed in synaptic vesicles (Neher and Sakaba, 2008); postsynaptically, a temporary increase of calcium at the level of the dendritic spines is responsible for activity-dependent synaptic plasticity (Zucker, 1999). Calcium ions are also involved in the regulation of gene transcription at the subcellular level (Lyons and West, 2011). As opposed to fMRI, calcium indicators provide an accurate and direct measure of neuronal activity with a high signal to noise ratio. More specifically, neuronal activity is measured as changes in the intracellular calcium concentration reflected by altered emitted fluorescence of the calcium indicator (Wachowiak et al., 2002).

Calcium indicators may be classified into three different types, each one characterized by distinct features: (1) the bioluminescent calcium indicator aequorin (Ohmiya and Hirano, 1996)*;* (2) chemical calcium indicators; (3) genetically encoded calcium indicators (GECIs). The bioluminescent calcium indicator aequorin is extracted from marine organisms. When calcium binds its calcium binding sites, aequorin undergoes a conformational change that results in the emission of a photon (Ohmiya and Hirano, 1996). This calcium indicator does not require external illumination, thus providing several advantages including avoidance of phototoxicity, photobleaching, autofluorescence, and induction of light-dependent biological processes. On the other hand, aequorin can perform only one photon emission per cycle and it has to be loaded into single cells using a micropipette. Moreover, aequorin does not represent the best choice in terms of quantum yield and protein stability. On the contrary, chemical calcium indicators (e.g., Fluo-3, Calcium Green-1, and Oregon Green BAPTA) can be loaded into the brain with different approaches such as multicell bolus loading (Stosiek et al., 2003; Schulz et al., 2012) and bulk electroporation (Nagayama et al., 2007). Moreover, there are several types of chemical calcium indicators with differential calcium affinity and spectral properties that can be simultaneously used. The last category, GECIs (e.g., GCaMP6, RCaMP) entered the stage of calcium imaging more than a decade ago and are currently widely used in neuroscience. They are characterized by high target specificity since they can exclusively be expressed in neuronal subpopulations of interest by means of viral constructs injected into brain areas of choice. Compared with chemical indicators (Paredes et al., 2008), they show reduced toxicity and are functional in neurons for longer periods of time, thus making them most suitable for longitudinal studies (Grienberger and Konnerth, 2012).

**FIGURE 3:**
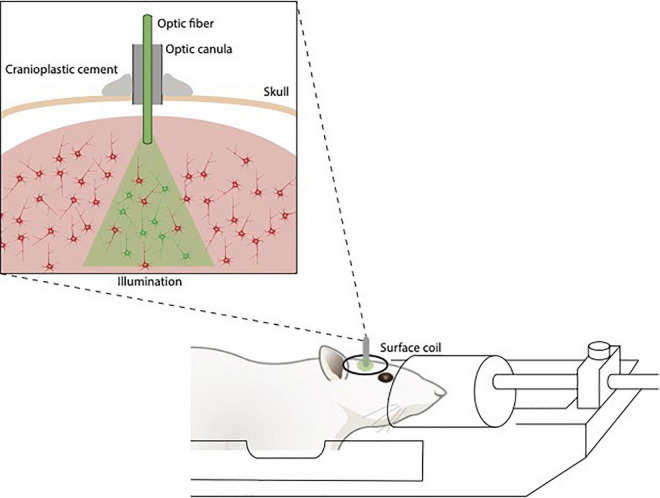
Illustration of fiberoptics/fMRI setup. The optic fibers are implanted above the opsin/calcium indicator-stained area, fixed to the skull, guided through the coil and connected to the stimulation/detection unit.

Calcium-based optical fiber photometry allows to investigate circuit-specific activity dynamics recording bulk changes in calcium fluorescence signals in genetically defined neurons in a relatively simple and artifact-free manner through an optical fiber implanted in the animal’s head (Schlegel et al., 2018). Proper fiber implantation is important for the success of the recordings. Because of the optical fiber, this technique allows to penetrate deeper brain regions (subcortical regions) in comparison with the older two-photon calcium imaging but at the same time it lacks in cellular resolution. To overcome this limitation, it can be alternatively combined with two-photon microscopy that, on the contrary, gives excellent spatial resolution (Resendez and Stuber, 2015). Two-photon-imaging in combination with novel calcium indicators (e.g., GCamp7) allows the recording of large networks of neurons (hundreds of cells) with high timing accuracy (single spike detection), yet in a complementary manner rather than simultaneously. Unlike electrophysiology, it does not require MRI sensitive components such as metal wires, hence it does not interfere with fMRI acquisition due to the lack of radiofrequency interference. fMRI and calcium-based optical photometry can be combined to correlate neuronal activity of specific neuronal networks to whole-brain activity and functional connectivity (Yu, 2017; Figure 3). Recently, MRI-compatible microscopes are being developed for optical readout during fMRI with the potential to provide a resolution superior to MR resolution and to highlight particular structures (Wapler et al., 2021).

To date, calcium-based optical fiber photometry represents an important milestone in the study of the correlation between neuronal activity and hemodynamics determining the BOLD signal.

The first paper reporting *in vivo* studies using calcium indicators in mammals (GCaMP3) was published in 2009 (Tian et al., 2009). Subsequently, many studies have been published using fiber photometry in combination with other techniques (e.g., fMRI, optogenetics), expanding the palette of multimodal imaging techniques. Schulz et al. (2012) demonstrated the feasibility of coupling fiber-optic calcium recording and fMRI to simultaneously acquire calcium-specific fluorescence emitted by chemical calcium indicators (OGB-1, Rhod-2, and Fluo-4) and BOLD signals in a forepaw stimulation paradigm and to reveal the complexity of the BOLD signal that involves not only neuronal but also glial activity. A similar approach was used by Schmid et al. (2016), who additionally manipulated neuronal activity with optogenetics. Independent of the type of stimulation (sensory or optogenetic), the calcium response was identical.

A major disadvantage of calcium dyes as opposed to GECIs is that longitudinal studies are more challenging due to their shorter decay time, loading approach and limited cell-type specificity. To overcome these limiting factors, it is possible to use GECIs and in particular, the improved generation of GECIs (Chen et al., 2013). Liang et al. (2017) used an improved version of GECIs, namely GCaMP6f, to demonstrate the feasibility of simultaneous calcium and BOLD signals recordings in order to elucidate the neural basis of the BOLD signal. The adopted approach was validated with a visual stimulation paradigm: upon visual stimulation, a robust increase of calcium and BOLD signals was detected. The importance of this study relies on the establishment of a setup that allows longitudinal and simultaneous GCaMP6-based fiber photometry and fMRI in rats, thus setting new perspectives to investigate the NVC. Recently, even more insights into the BOLD signal have been revealed by coupling fiber-optic calcium recordings to fMRI. He and colleagues have developed single-vessel resting-state fMRI to delineate vessel-specific fluctuations in the BOLD signal (He et al., 2018). Combining this technique with simultaneous intracellular calcium recordings, they have been able to investigate whether neuronal activity is correlated with fluctuations in the cerebral vasculature. This method has allowed to determine the temporal dynamic features of neurovascular fluctuations and to define the spatial distribution of these fluctuations in the BOLD signal in the neurovascular network. The data show that neural activity and intrinsic vascular fluctuations are correlated. Although emerging evidence suggests that the hemodynamic response function (HRF) can vary by brain region and species, a single, canonical, human-based HRF is widely used in animal studies. Therefore, the development of flexible, accessible, brain-region specific HRF calculation approaches is paramount as hemodynamic animal studies become increasingly popular. Chao et al. calculated empirical HRFs for various rat brain regions, by simultaneously measuring neuronal activity *via* GECI GCaMP6f, local cerebral blood volume (CBV) from intravenous Rhodamine B dye, and whole brain CBV *via* fMRI with the Feraheme contrast agent. Empirical HRFs were calculated with GCaMP6f and Rhodamine B recordings from rat brain regions during resting-state and task-based paradigms, resulting in each HRF faster and narrower than the canonical HRF and no significant difference between these regions. When used in general linear model analyses of corresponding fMRI data, the empirical HRFs showed better detection performance than the canonical HRF (Chao et al., 2022).

It is evident that a multimodal approach in which fMRI is simultaneously coupled with calcium optical fiber recording is of exceptional utility and validity. Last but not least, the use of simultaneous fMRI and GCaMP-mediated Ca^2+^ optical fiber recording has allowed to discover the role of the astrocytic Ca^2+^ signal in the modulation (vasodilatation/constriction) of the neurovascular network and its influence on thalamic regulation of cortical states. Interestingly, it has been found that sensory-evoked and intrinsic astrocytic Ca^2+^ signals were, respectively, coupled with positive and negative BOLD signals, giving rise to specific NVC events (Wang et al., 2018). In this regard, the work of Lake et al. is of interest as well, highlighting MR-compatible optical calcium imaging approached by concurrent wide-field optical and functional MRI (Lake et al., 2020). Pais-Roldán et al. (2020) went a step further to combine both simultaneous fMRI and fiber-optic calcium recordings with pupillometry to enable tracking of brain-state dependent pupil dynamics and identifying unique cross-scale neuronal dynamic patterns under anesthesia. This study suggested that the pupil dynamics observed during anesthesia in rats are directly linked to the global fMRI signal fluctuation, as well as to the cortical population activity observed as neuronal calcium transient oscillations, and perfectly exemplifies the successful combination of imaging modalities. A recent study consisting fiber-based optical recordings in animals expressing the genetically encoded calcium sensor Twitch-2B combined with fMRI elucidated the pitfall of hemodynamic artifacts and how to correct them (Lambers et al., 2022). The authors identified prominent effects of hemodynamic artifacts in the timecourses of fluorescence and fMRI signals and applied an MR parameter-based correction algorithm to yield results consistent with expected signal changes.

In recent years, the development of multimodal nanoprobes has been growing. An example of these nanostructures is a bimodal system based on quantum dots and low molecular weight Gd^3+^ chelates, prepared for MRI and optical analyses (Albuquerque et al., 2021). Non-covalent nanosystems range in size from 7 to 160 nm, and a relaxivity value of 4.5 (at 1.5 T) to 31.5 (at 35 MHz) mM^–1^s^–1^ per Gd^3+^. Covalent nanosystems can range from 8 to 45 nm diameter, and relaxivity values from 1.6 (at 3 T) to 24 (at 1.5 T) mM^–1^s^–1^ per Gd^3+^. It can be concluded that the preparation method is independent of both the final nanoprobe size and their relaxivity values. Studies about the nanoprobe stability and inertness in the presence of endogenous metals in mammals are still required.

## Neural perturbation: Combining magnetic resonance imaging with neuronal excitation or inhibition

### Electrical stimulation

While electrical neural activity can be probed by means of electrophysiology as described above, targeted brain regions can also be stimulated through electrodes in a technique known as deep brain stimulation (DBS). DBS has developed over the last 20 years into a highly effective evidenced-based treatment option for neurological disorders. Moreover, it has become a fascinating tool to provide illustrative insights into the functioning of brain networks. Similar to electrophysiological recordings, brain stimulation is achieved by means of stereotactically implanting electrodes into specific targets in the brain, and delivering constant or intermittent electric currents from an implanted battery source (Gonzalez-Escamilla et al., 2020).

Non-invasive brain circuit tracing, using fMRI, allows the exploration of direct clinical effects of DBS. However, fMRI studies on patients implanted with DBS electrodes are limited because of safety concerns and imaging artifacts (Tagliati et al., 2009; Kahan et al., 2015), therefore, animal models typically are used with these aims. A technique by Tolias et al. (2005) to record brain activity using the blood oxygen level dependent (BOLD) signal while applying electrical microstimulation to the primate brain, demonstrated its utility in uncovering *in vivo* functional connectivity maps.

In non-human primates, subthalamic nucleus (STN)-DBS increases BOLD activation in the sensorimotor cortex, supplementary motor area, caudate nucleus, pedunculopontine nucleus, cingulate, insular cortex, and cerebellum (Min et al., 2014). Similarly, increased BOLD responses in motor, somatosensory, and cingulate cortices to STN- and internal global pallidus (GPi)-DBS are found in rats (Lai et al., 2014). In pigs, DBS targeting of the STN and the entopeduncular nucleus, the non-primate analogue of the primate GPi, induces increased BOLD activity in the ipsilateral sensorimotor network, including the premotor, primary motor, and primary somatosensory cortices, as well as dorsolateral prefrontal and anterior cingulate and insular cortices. The network activations showed differential, target-specific, non-motor network effects (Min et al., 2012).

Neurons can be stimulated electrically through metal or MRI compatible glass electrodes, but electrical stimulation is nonspecific regarding cell-type and allows to directly trigger neuronal activity only in single or a small group of neurons dependent on the position of the stimulating electrode.

### Optogenetically modulating neuronal activity using light

In addition to neuronal activity measurements, light can also be used to modulate neuronal activation. More promising than the electrical stimulation described above is the relatively new technique known as optogenetics, which guarantees higher genetic, temporal and spatial specificity (Nagel et al., 2003; Zhang et al., 2007). The use of light is paving the way to a more specific and effective control and investigation of neuronal networks’ activity.

Optogenetics represents a technique of choice to modulate neuronal activity using light. Its implementation results from the need to establish a method that can be used to pinpoint how different neuronal subtypes cooperate to carry out thoughts, feelings, movements and how their activity is affected in brain pathology. Optogenetics has revolutionized the field of neuroscience because it allows, as opposed to classical electrical brain stimulation techniques, a real-time direct optical manipulation of specific neuronal subtypes, ensuring both excellent spatial and temporal resolution in various animal models (Yizhar et al., 2011). For this technique, target cells are genetically modified to express light-activated ion channels like channelrhodopsins (ChR), halorhodopsin and light-gated ionotropic glutamate receptors (Lin, 2011). In this way, their activity can be triggered or inhibited by light on a millisecond time scale. Microbial opsins can be selectively expressed in target neurons using viral vectors (Yizhar et al., 2011). Optogenetics does have some possible caveats that should be taken into account when planning optogenetic experiments (Guru et al., 2015): (1) opsins’ expression and light delivery is not uniform in all targeted neurons; (2) fiber placement is of importance: if the fiber is just slightly displaced, the success of the experiment could be impeded; (3) the delivered light indiscriminately modulates all the neurons of the targeted neuronal population, shadowing individual neuronal activity patterns; (4) opsins’ expression can alter the physiological activity of neurons, including their cellular machinery due to the expression of “additional” ion channels in the cellular membrane; and (5) the specificity of light excitatory modulation can be impaired because direct delivery of light on the axonal membrane can also result in the excitation of terminals projecting to other brain areas which are not of interest.

Although the history of the methodological development can be traced to the 1970s, one of the most important milestones in the implementation of this technique was not achieved until 2005, when Karl Deisseroth’s group conducted the expression of channelrhodopsin-2 (ChR2) in neuronal cells (rat hippocampal cultured cells) to identify light-evoked neuronal activity (Boyden et al., 2005). Nowadays, this breakthrough technique permits to achieve a virtually complete neural circuit mapping, thus providing a causal link between brain activity and behavior in healthy and pathological conditions (Tye and Deisseroth, 2012). From its appearance until now, implementation of optogenetics has been made much easier thanks to the growing literature dealing with several technical aspects of this technique such as opsins’ choice, light source (lasers, LEDs) and delivery (intensity, depth of illumination), genetic targeting specificity and stimulation/inhibition protocols.

Optogenetics can be combined with fMRI resulting in what is known as optogenetic fMRI (opto-fMRI). Opto-fMRI combines the precision of optogenetic manipulation and the high spatial resolution at the whole-brain level of fMRI that enable the examination of activity and functional connectivity of specific neural networks at the whole-brain level without electromagnetic interference with the radiofrequency signal. Opto-fMRI allows to indirectly measure neuronal activity in the whole brain at high spatial resolution, independently from the site of stimulation (Lee et al., 2010). Specifically, this method allows to determine the effects of activation/inhibition of a specific target neuronal population on whole-brain activity and functional connectivity. To combine both techniques, some precautions should be taken. Importantly, the optical fiber and implanted cannula used to deliver light into the brain must be MRI compatible. Further, the laser power and fiber implantation have to be carefully established to avoid false BOLD responses. In fact, a negative BOLD response has been observed in the area under the fiber tip during high laser power stimulations (Desai et al., 2011). In 2013, Christie and colleagues have again noticed that fMRI responses are influenced by laser power, which acts as a possible confounding factor in opto-fMRI studies. Indeed, prolonged (30s) blue light exposure of naïve brains of dead rats has caused a temperature increase and has impacted the MR signal in terms of temperature dependent changes in the relaxation times T1 and T2* (Christie et al., 2013).

While taking these caveats into consideration, some important research has been done using opto-fMRI. Lee et al. (2010) published a paper that has proven that optogenetics can be used together with high-field MRI to study the BOLD signal evoked by optical stimulation of specific brain areas in anesthetized rats. In particular, opto-fMRI was used to explore the role of local excitatory neurons in the induction of the BOLD signal. To this aim, ChR2 was expressed under regulation of the Ca2+/calmodulin-dependent protein kinase II (CAMKII) promoter in the primary motor cortex (M1) of adult rats by means of an AAV vector. Upon optical stimulation, light-activated neurons elicited a BOLD response not only in the illuminated area (M1) but also in the non-optically stimulated thalamus. This study is important not only for its findings related to the nature of the BOLD signal but also because it underlines crucial features characterizing opto-fMRI. The most relevant is the opportunity to globally map the effect of the manipulation (light stimulation) of a specific neuronal population on the whole-brain activity in rodents, rendering information about the networks recruited by local neuronal populations.

Abe et al. (2012) used opto-fMRI in a 2012 study to explore the neuronal connectivity of the hippocampal formation utilizing a rat strain that expressed ChR2-venus protein. After optical stimulation at the dentate gyrus, they obtained a BOLD response not only in the dentate gyrus, but also in CA3, and they also detected the longitudinal-axis activation of the hippocampal formation. The authors state that these results may accelerate the search for dynamical functional brain mapping, as well as increasing our understanding of hippocampal networks. More importantly though, it further establishes opto-fMRI as a powerful tool for neuroscience.

More recently, Christie et al. (2017) conducted the first three-dimensional study of spatial correlations of NVC using single pulse opto-fMRI, a technique based on the delivery of only one brief and short light stimulus. This approach ensures (1) a well-defined modulation of the volume of activated neurons in a determined brain region, spatially confining the recruitment of neural activity; (2) minimization of vascular and neuronal adaptation; and (3) prevention of heating artifacts. It provides a unique view of the spatial features of the NVC. ChR2 was expressed in cortical glutamatergic neurons of anesthetized rats, which then were illuminated with a single 10 ms pulse of blue light (inter-stimulus interval 20 s) of gradually increasing intensities to manipulate the spatial extent of the stimulus. Simultaneously, a 3D-mapping of functional hyperemia was done in order to estimate the volume of neural and hemodynamic correlations. As previously reported (Leithner and Royl, 2014), the spatial extent of the BOLD response is way more extended than the estimated volume of neuronal activation. However, a major limitation was that the spatial extent of neuronal activation was not directly measured but only estimated on the basis of the scattering and absorption features of blue light in the brain. A very recent study by the Grandjean group used opto-fMRI to show enhanced fMRI signal within several projection areas following optogenetic activation of the entorhinal cortex 3xTgAD mice (Mandino et al., 2022).

A major hurdle for making full use of the versatility of optogenetics, and thus of opto-fMRI, and GECI, is the application in species with low or no availability of transgenic lines in species such as primates (Gerits and Vanduffel, 2013). The highly flexible Cre-LoxP/adeno-associated viral (AAV) vector delivery method is usually dependent of transgenic lines expressing Cre recombinase under the desired cell-type-specific promoter, or alternatively of co-injection of an AAV vector expressing Cre recombinase under the desired cell-type-specific promoter (Gompf et al., 2015). Large libraries of such transgenic lines are available for rat and mouse models (Han, 2012), yet for other animal models there is no comparable availability, and consequently, opto-fMRI applications in these settings generally make use of more unwieldy custom lentiviral vectors, as well as close consideration of the trade-off between promoter length and expression characteristics.

One key feature of optogenetics is the ability to selectively modulate cells closely relevant to specific neurotransmitter systems. Prominent studies of neurochemical systems with fMRI include dopamine modulation (Decot et al., 2017; Lohani et al., 2017; Ioanas et al., 2022a), with findings suggesting that large-scale brain-wide activity dynamics, measured across many distinct neuroanatomical regions, are also directly or indirectly regulated by the activity of VTA dopaminergic neurons *in vivo* (Decot et al., 2017), and that mesolimbic and non-limbic basal ganglia dopamine circuits are functionally connected (Lohani et al., 2017). Ioanas et al. (2022a) present a comprehensive assay producing the first whole-brain opto-fMRI map of dopaminergic activation in the mouse, and show that VTA dopaminergic system function is consistent with its structural VTA projections.

#### New frontiers in small animal imaging: Simultaneous fiber-optic calcium recordings and opto-functional magnetic resonance imaging

While technically challenging, it is possible to combine fiber-optic calcium imaging together with opto-fMRI to simultaneously modulate genetically targeted neuronal populations, record their neuronal activity and acquire whole-brain activity and functional connectivity data. This would be utterly difficult with electrophysiology instead of calcium imaging/optogenetics because it does not allow to record or modulate neuronal activity from hundreds of genetically identified neurons at the same time. As previously discussed for opto-fMRI and fiber-optic calcium recordings combined with fMRI, all the set-up components must be MRI compatible or at least positioned outside the scanner room. Importantly, both opsins and calcium indicators need to have non-overlapping spectra in order to avoid potential cross-talk (Akerboom et al., 2013). If optogenetics and fiber-optic calcium recordings are performed in the same brain area using the same excitation wavelength, the detecting fiber can be saturated by fluorescence. This phenomenon can be prevented in two ways: by using different wavelengths for opsin and calcium indicators or by spatially separating the area of the opsin’s excitation and the region of neuronal activity detection (e.g., the light used to excite the opsin is delivered in the medial septum and the detection of calcium indicators’ fluorescence is done in the hippocampus). If the second solution is adopted, the excitatory fiber and the detecting fiber have to be precisely positioned to ensure that excitation and detection happen in their exact locations. Further, before starting the experiment, the laser power has to be defined in order to avoid tissue heating and false MRI signal. The stimulation duration is important as well: while short pulses (in the order of millisecond) are sufficient to activate opsins such as ChR2, a longer stimulation is required for BOLD measurements. It has been demonstrated that a BOLD response can be evoked by a single 10 ms pulse of excitatory light (Christie et al., 2017) or with consecutive shorter pulses (1 ms) lasting several seconds (Albers et al., 2018). Chen X. et al., 2019 verified the multi-modal fMRI platform in combination with both optogenetic stimulation and simultaneous Ca^2+^ recordings to map optogenetically-driven single-vessel fMRI with concurrent neuronal calcium recordings in the rat hippocampus. By implementing simultaneous optogenetic single-vessel fMRI and optical fiber Ca^2+^ recordings, distinct hemodynamic spatiotemporal patterns across the hippocampal vasculature could be directly characterized based on concurrent neuronal Ca^2+^ signals for the first time. It is worth noting that in labs with a specialized infrastructure, it is possible to monitor the positioning of the optical fiber with MRI feedback to ensure that the recording or stimulation is done in the optimal place. In this regard, Chen et al. described an MRI-guided robotic arm as a flexible positioning system to assist optical fiber brain intervention in real time in a study involving optogenetic fMRI with concurrent Ca2+ recording (Chen Y. et al., 2019).

### Perturbation of neural activity using chemogenetics

Altered neural signaling can also be achieved using a chemogenetic approach, the most commonly used being Designer Receptors Exclusively Activated by Designer Drugs or DREADDs. DREADDs are extremely useful research tools that allow a targeted manipulation of certain pathways in the brain. DREADDs are genetically modified G protein coupled receptors (GPCRs) that only respond to an exogenous drug and not to any endogenous molecule, activating one of three major GPCR signaling pathways, ultimately leading to activation or inhibition of the DREADD-expressing cells. Using DREADDs, one can unravel the specific role of different pathways in the brain. Compared with optogenetics, DREADDs have the advantage that they can be activated by systemic administration of a pharmacological compound and that they have less temporal restrictions, which renders them more attractive for therapeutic purposes. Although the temporal precision and control of chemogenetics is lower than optogenetics, it has been proven to be an invaluable tool in modern neuroscience allowing cell-type-specific modulations, bidirectional control of cells, and mapping of functional networks.

*In vivo*, DREADDs expression in specific cell types is typically achieved by using transgenic mouse models or focal viral vector injections targeting selected brain areas. Various transgenic mouse models have been developed to specifically express DREADDs in selected cell populations by using specific promoters (Guettier et al., 2009; Farrell et al., 2013), the transcriptional Tet-On/Off system (Alexander et al., 2019) or the Cre-recombinase system (Zhu et al., 2016). On the other hand, DREADDs can also be expressed using a completely virally mediated method. In this case, a viral construct is intracranially injected and the cellular transduction and expression of the DREADDs is determined by the viral vector’s serotype (of genome and capsid) and promoter gene (Aschauer et al., 2013). After successful expression of the designer receptors, they can be activated by administration of a designer drug, such as clozapine-*N*-oxide [CNO, Armbruster et al. (2007)] or JHU37160 (Bonaventura et al., 2019).

Our lab has recently made progress studying targeted cholinergic stimulation in rodents using pharmacological fMRI (phMRI) (Peeters et al., 2020a,c). The study of this chemogenetic manipulation with phMRI is a method without any interference to the MRI hardware, as no external hardware is necessary for targeted brain stimulation. In literature, DREADDs stimulation combined with MRI has been used to capture the effect of selective activation of certain neuronal populations. Baslow et al. (2016) used functional magnetic resonance spectroscopy to evaluate alterations in local neuronal *N*-acetyl-aspartate as well as a BOLD fMRI to assess changes in neuronal activity. In this study, a viral delivery method was used to express excitatory hM3Dq-DREADDs in medial prefrontal cortex neurons. CNO injection elicited a focal decrease in the *N*-acetyl-aspartate signal and an increased BOLD response. In addition, local field potentials were recorded as a direct measure of neural activity and confirmed that CNO could increase neural activity through activation of hM3Dq, supporting the utility of DREADDs *in vivo* (Baslow et al., 2016). Roelofs et al. expressed hM3Dq receptors in the mesolimbic and mesocortical pathway in rats. Significantly increased BOLD responses were observed in the ventral tegmental area, nucleus accumbens, and medial prefrontal cortex after CNO injection. DREADDs stimulation not only induced activation in the mesocorticolimbic regions but also drove BOLD response alterations at the whole-brain level. In contrast to the altered neural activity, the functional connectivity remained stable after CNO injection (Roelofs et al., 2017). Giorgi et al. (2017) highlighted the advantage of chemogenetics combined with fMRI to causally link the activity of specific neuronal populations with whole-brain functional activity. The stimulation of serotonergic neurons upon CNO injection in transgenic mice expressing excitatory DREADDs in serotonergic neurons, resulted in a significantly increased relative cerebral blood volume in connected regions encompassing corticohippocampal and ventrostriatal regions while the administration of the selective serotonin reuptake inhibitor citalopram globally decreased the relative cerebral blood volume (Giorgi et al., 2017).

Another neurochemical system studied with a chemogenetic-fMRI approach is the norepinephrine system. Zerbi et al. (2019) used a mouse model to study the effect of locus coeroleus (LC) stimulation on large-scale functional connectivity and showed that LC activation rapidly interrupts ongoing behavior and strongly increases brain-wide connectivity, with the most profound effects in the salience and amygdala networks. Oyarzabal et al. (2022) reported that chemogenetic-induced tonic LC activity decreased cerebral blood volume (CBV) and glucose uptake and increased synchronous low-frequency fMRI activity within the frontal cortices of the DMN.

For a full review of the combination of chemogenetics with *in vivo* imaging modalities, we refer to Peeters et al., 2020b.

In contrast to optogenetics, chemogenetics allow for remote and longer lasting activation/inhibition of neuronal activity without the need for an optic fiber implantation. *In vivo* imaging will allow non-invasive, longitudinal monitoring of disease progression and therapy response in patients that will undergo DREADD-based neuromodulatory therapy.

## Conclusion and future directions

**FIGURE 4:**
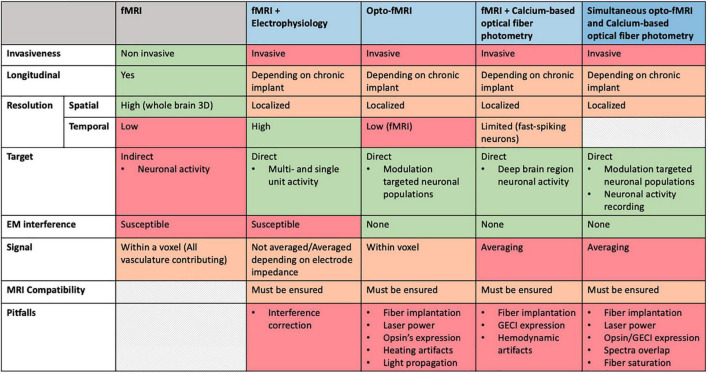
Comparative overview of specifications for multimodal imaging approaches.

Advancements in neuroscience and specifically in the investigation of NVC are essentially owed to the recent technical improvements developed in the field of multimodal MRI. Above all, a major role is played by the development and combination of techniques such as fMRI, optogenetics and fiber-optic calcium imaging that are able to efficiently manipulate not only individual cells, but also neuronal networks at a whole-brain resolution. Figure 4 summarizes strengths and caveats that need to be considered when combining techniques in a simultaneous recording setup.

In short, if the main focus of the experiment is to acquire direct and not averaged recordings of neuronal activity of single neurons or at least a limited number of them, electrophysiology still remains the best choice, whereas if the scientist aims to investigate/modulate neuronal activity of genetically identified neuronal types at a network level, optical techniques are recommended. However, as is the case with any newly developed methodology, one must also be wary of the caveats. Recently, Christophe Bernard reviewed some confounding factors that should be taken into account when interpreting optogenetic effects (Bernard, 2020), such as brain dynamics, cell dynamics, possible perturbations by inserted channelrhodopsins, possibility of the light perturbing native proteins and modifications triggered by the viral vectors. We have to always be aware of what this means for data interpretation.

Today’s systems neuroscientists face a choice. While the animals they study engage in some behavioral task, how should they monitor neural activity? Should they use electrodes? Should they introduce into their neurons of interest a molecule that will signal activity and image it? Should they monitor whole-brain activity or use localized techniques? The main goal of multimodal MRI is to simultaneously combine fMRI with other different methods to take advantage of their technical strengths while compensating for possible weaknesses. It represents a powerful tool to investigate the link between neuronal activity and vascular network dynamics from single cell or circuit level to the whole brain with a multiscale approach. Electrophysiology and MRI have been considered the opening chords of a revolution characterized by a shift from electrons to photons and from the application of single techniques (e.g., electrophysiology, fMRI) to the simultaneous combination of fMRI with techniques able to modulate and measure neuronal activity.

Although the described techniques complement each other well, additional technical improvements are critical. These advancements may pave the way for longitudinal brain imaging studies providing single-cell and network resolutions as well as specific control of neuronal activity. For example, a huge effort has been done to further improve the optogenetics’ toolbox as well as the variety of calcium indicators. In fact, many variants of opsins (Lin, 2011; Lin et al., 2013) and calcium dyes (Zhao et al., 2011) have been developed. Currently, attention is focused on the development of red-shifted calcium indicators (Wu et al., 2013; Rose et al., 2014) that, unlike the blue emitting indicators: (1) can be expressed together with ChR2 or blue-emitting calcium indicators allowing multi-labeling without the risk of spectra overlapping; and (2) are characterized by reduced light scattering, thus giving higher tissue penetration with reduced excitation power. However, kinetics and spectral response remain to be improved.

A new frontier in deep brain stimulation using light is presented in the paper of Chen and colleagues where they have demonstrated the ability to perform deep brain stimulation using upconversion nanoparticles (UCNPs) (Chen et al., 2018). UCNPs are specialized nanoparticles able to upconvert the absorbed near-infrared (NIR) light to visible blue light. Innovative is the attempt to solve the invasiveness of inserting a laser in the brain to activate light-sensitive channels: targeted deep brain areas can be non-invasively stimulated through tissue-penetrating NIR light shined from outside the brain. Further, transgenic rodents have been generated to guarantee the cell-type specific expression of opsins (Zhao et al., 2011; Madisen et al., 2012) as well as of GECIs (Dana et al., 2014; Sato et al., 2015). The use of transgenic lines helps to overcome limitations characterizing the viral expression systems where gene delivery is mediated *via* brain injection of a viral vector, such as limited labeling of target neurons and problematic access of the virus to determined brain areas. Additionally, a more stable, reproducible and homogeneous expression over a longer period of time can be achieved. Even if these techniques are continuously improved as new technologies arise, there are still a few technical issues affecting multimodal imaging that need to be addressed in the near future to allow remarkable steps forward in the field of small animal imaging.

## Author contributions

GK led the review. LK finalized the manuscript, undertook part of the systematic review, and designed the figures. SE drafted the first manuscript and undertook part of the systematic review. SM made critical comments on the manuscript and reviewed the final report. All authors contributed to the article and approved the submitted version.

## Conflict of interest

The authors declare that the research was conducted in the absence of any commercial or financial relationships that could be construed as a potential conflict of interest. The handling editor declared a past co-authorship with authors GK and SM.

## Publisher’s note

All claims expressed in this article are solely those of the authors and do not necessarily represent those of their affiliated organizations, or those of the publisher, the editors and the reviewers. Any product that may be evaluated in this article, or claim that may be made by its manufacturer, is not guaranteed or endorsed by the publisher.
